# Breast tissue ablation with irreversible electroporation in rabbits: A safety and feasibility study

**DOI:** 10.1371/journal.pone.0181555

**Published:** 2017-07-21

**Authors:** Wenlong Zhang, Wanning Wang, Wei Chai, Xiaomei Luo, Jiannan Li, Jian Shi, Liqi Bi, Lizhi Niu

**Affiliations:** 1 Department of Hematology and Oncology, China-Japan Union Hospital of Jilin University, Changchun, China; 2 Department of Nephrology, First Hospital of Jilin University, Changchun, China; 3 Department of Gynecology and Obstetrics, The First Hospital of Jilin University, Changchun, China; 4 School of Medicine, Jinan University, Guangdong Province, Guangzhou, China; 5 Department of General Surgery, The Second Hospital of Jilin University, Changchun, China; 6 Department of Rheumatology and Immunology, China-Japan Union Hospital of Jilin University, Changchun, China; 7 Fuda Cancer Hospital, Jinan University School of Medicine (Guangzhou Fuda Cancer Hospital), Guangzhou, China; Consiglio Nazionale delle Ricerche, ITALY

## Abstract

**Background and aim:**

Irreversible electroporation (IRE) was confirmed to control several solid tumors effectively in vivo. Our preclinical study aimed to assess the feasibility and safety of IRE in the breast of rabbit.

**Methods:**

Thirty New Zealand white rabbits were randomly divided into 3 groups of 10 rabbits (control group, IRE group A, and B). Two mono-electrode needles were inserted into the breast tissue by percutaneous puncture. Electrocardiogram and vital signs were monitored before, during, and after ablation. Histopathology, immunohistochemistry, and transmission electron microscopy were examined at 0 hours, 12 hours, 24 hours, 4 days, 7 days, 14 days, and 28 days after ablation.

**Results:**

All the rabbits survived the procedure with no significant adverse effects. Intra-operative ventricular arrhythmias occurred in 1 rabbit from IRE group B and was immediately relieved after ablation. Reversible subcutaneous hemorrhage was observed in 8 rabbits from IRE group A and 7 rabbits from IRE group B. No skin was burnt, however, pectoralis major muscle injuries were found in all rabbits. Histopathological and ultrastructural examination revealed the coexistence of cell necrosis and apoptosis. HE, TUNEL, and Masson staining revealed breast tissue injury and the recovery of damage by fibrous tissue and granulation tissue. Notably, the structures of mammary gland lobules and interstitial components of the breasts were well preserved.

**Conclusions:**

Our study suggests that IRE destroys breast cancer while effectively preserving the skin, the structure of mammary gland lobules, and interstitial components. IRE may be a promising technique to locally control breast cancer and to maintain the esthetic of the breast.

## Introduction

Breast cancer, currently the second leading cause of malignant tumor deaths in the United States, is a major public health concern [1]. A survey reported by Siegel et al. predicted that 231,840 new cases and 40,290 new deaths were expected in the United States in 2015 [2]. For breast cancer patients from stages I to III, surgery is the standard recommended treatment. Furthermore, according to the molecular subtype, preoperative and/or postoperative adjuvant therapy may also be needed in order to realize the goal of individualized treatment [3]. Owing to breast asymmetry after surgery, some patients may not be satisfied or may become anxious, and this may lead to poor psychological well-being or the financial burden of breast cosmesis [4, 5]. In addition, with the popularity of screening for breast cancer, more patients can be diagnosed at an early stage or at smaller tumor sizes than before [6–8], suggesting that minimally invasive treatment and better esthetic outcomes may come to be expected for early-stage breast cancers [9–11]. Furthermore, not all patients can tolerate surgery, especially older breast cancer patients with a variety of comorbidities [12, 13]. Therefore, minimally invasive treatment would be a promising approach for early-stage and elder patients with breast cancer [11].

Unlike conventional thermal ablation methods, irreversible electroporation (IRE) can create irreversible membrane pores and induce cell death by delivering high voltage electrical impulses, leading to cell dysregulation and death [14]. Moreover, IRE ablation may protect the integrity of vital vascular, nerve, and cytoskeleton structures as much as possible [15]. It has been widely used in the treatment of unresectable pancreatic cancer, hepatocellular carcinoma, and prostate cancer, and can not only delay the progression of the disease, but also maintain residual organ function [16–18]. Therefore, taking tumor ablation and breast cosmesis into account, IRE is expected to become the ideal choice for minimally invasive treatment of breast cancer.

Currently, the safety of IRE in human cancers has been studies in some reports, such as in patients with advanced malignancy [19]. However, reports on IRE for breast cancer remain limited. Robert *et al*. firstly performed in vitro experiments with MDA-MB-231 human breast carcinoma cells and suggested that IRE may serve as a successful treatment for breast cancer [20]. Though the feasibility of IRE for treating breast cancer has been studied in some researches [21], it is worth noting that IRE is still a relatively new field for breast cancer, and many issues remain to be addressed. Here, we established a systematic investigation regarding the safety and feasibility of IRE in breast cancer based on evaluation by histopathology, electron microscopy and immunohistochemistry and the influence on the appearance of the breast after IRE.

## Materials and methods

### Experimental design

**Fig 1:**
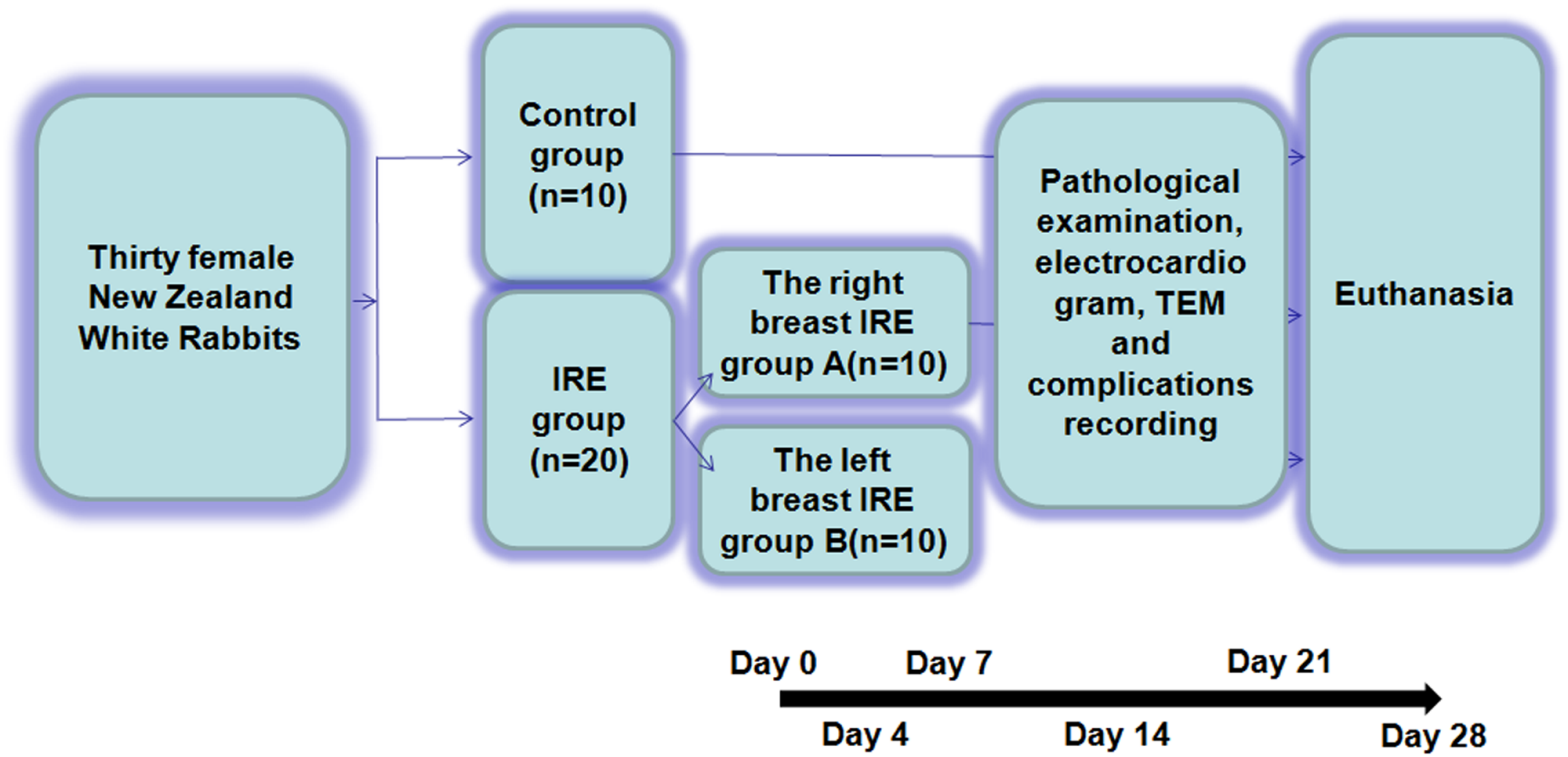
Flow chart exhibiting the design and process of this study. IRE, irreversible electroporation; TEM, transmission electron microscope.

Our study design is summarized as a flow chart in Fig 1.

### Experimental animals

Thirty certified healthy female New Zealand White Rabbits weighing between 3.0 and 3.5 kg (provided by the animal experiment center of Southern Medical University, Guangzhou, China) were employed in this study. Our research was approved by the Research Animal Care and Use Committee of South Medical University, and our experimental process complied with the Guide for the Care and Use of Laboratory Animals.

### IRE ablation system

**Fig 2:**
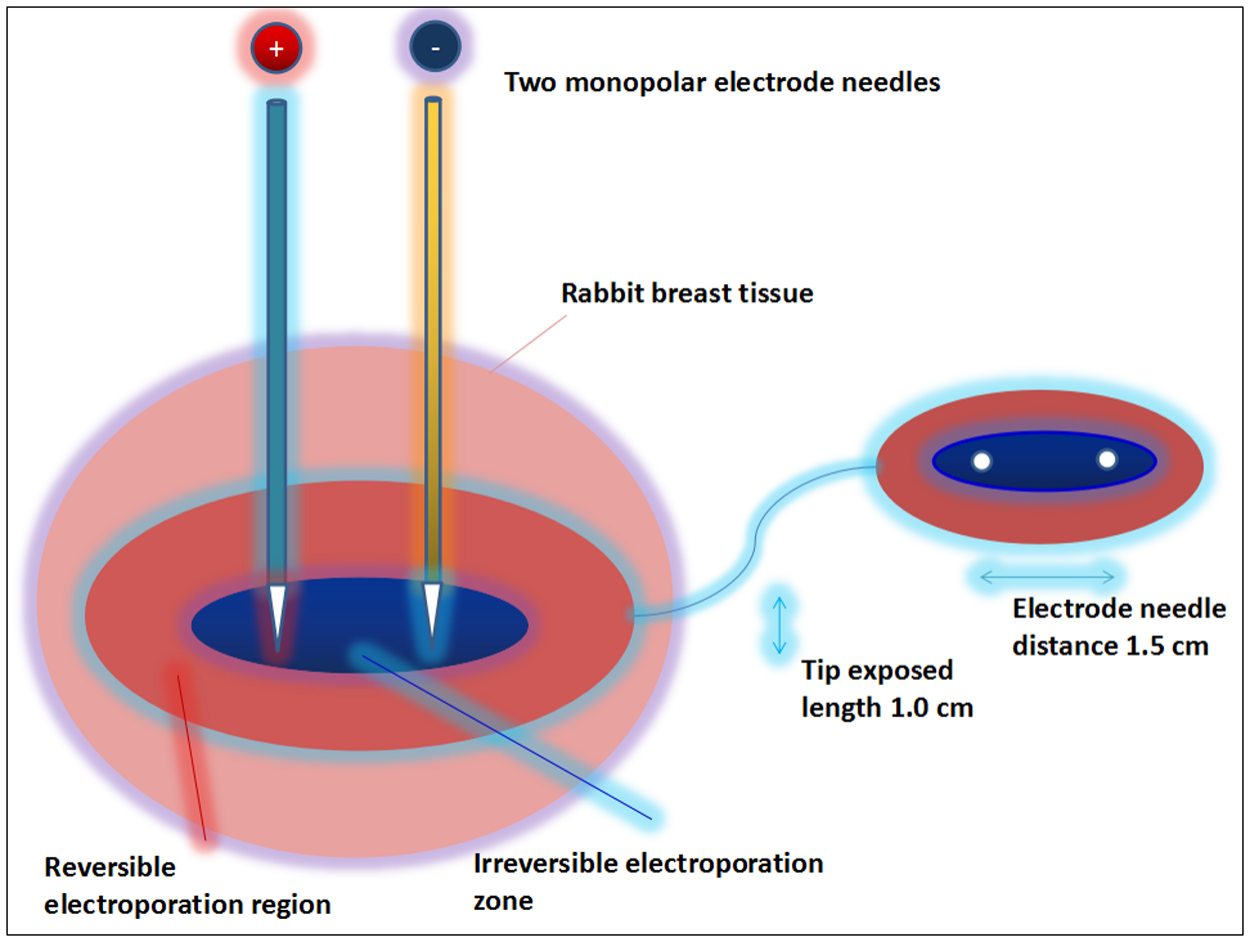
Schematic diagram of IRE ablation and pre-configured parameters.

Our experiments were conducted with a current pulse-generating device (Nanoknife, AngioDynamics^®^). Two monopolar electrode needles were used in the ablation process: one positive electrode probe launched electrical pulses and another negative probe served as the current circuit. Given the nipple size and chest wall thickness of the rabbits, the exposure depth of the electrode was set at 1.0 cm, and the distance between the electrodes was set at 1.5 cm (Fig 2). In order to investigate the safety and effects based on the most severe cases of tissue damage, we used the maximum threshold of the electric field strength generated by our device. For the IRE protocol, we treated the rabbits one cycle with 90 pulses, each lasting 70 μs, with an electrical field magnitude of 2000 V/cm.

### Experimental methods

To avoid muscle spasms caused by each pulse, adequate muscle relaxation is a prerequisite for successful electrical ablation. Thirty female New Zealand White Rabbits were administrated with limb injection of ketamine (Fujian Gutian Pharmaceutical Co., China, 75–100 mg/kg), phenergan (Suicheng Pharmaceutical, China, 1.0 mg/kg), and atropine (Jinyao Pharmaceutical, China, 5 μg/kg) before percutaneous puncture. The rabbits then received tracheal intubation and mechanical ventilation. The parameter for the small-animal ventilator was set as follows: tidal volume 20 ml, respiratory ratio 1:2, and respiratory frequency 30 times per minute. To obtain the ideal state of muscle relaxation, intramuscular injection of vecuronium bromide (Chengdu Tiantaishan Pharmaceutical, China) was given 10–15 min before ablation with a loading dose of 0.12 mg/kg. Complete muscle relaxation was essential; if required, we added 0.12 mg/kg vecuronium bromide during the minimally invasive operation.

The first breast on the right side of the rabbits from IRE groups A was disinfected with iodophor before ablation and exposed with an aseptic hole-towel. The first breast on the left side of the rabbits from IRE group B was treated following the same procedure. Two mono-electrode needles were inserted into the breast tissue in parallel with a tip distance of 1.5 cm. Other parameters were set as previously described. After one cycle of electrical ablation, we continued mechanical ventilation until spontaneous breathing recovered. To prevent postoperative infection and pain in the site of ablation, 40 U/ml of penicillin (CHINO Pharmaceutical Co. Ltd, China) and Metacam (Boehringer Ingelheim, France, 1–2 mg/kg) was injected once a day for 3 days. The rabbits in the control group underwent the same anesthesia and needle insertion process as described above without any release of current.

### Histopathology and ultrastructure monitoring

In order to understand the changes that normal breast tissues undergo after IRE ablation, rabbit breast tissue were embedded in paraffin, fixed on a slide, and stained with hematoxylin & eosin (HE), TUNEL (transferase-mediated deoxyuridine triphosphate-biotin nick end labeling), and Masson (masson's trichrome stain) before IRE and on days 1, 4, 7, 14, and 28 after IRE. In order to study ultrastructural changes following IRE, breast tissue in the control group and the IRE groups were soaked in 2.5% glutaraldehyde for transmission electron microscope (TEM) examination.

### Statistical analysis

Statistical analyses were carried out using GraphPad Prism 5 (GraphPad Software, San Diego, California). Student’s *t*-test was used to analyze the difference between two groups, with *p* values < 0.05 considered statistically significant, and *p* values < 0.01 or 0.001 indicating a highly significant difference.

## Results

### Animal safety and complications

**Fig 3:**
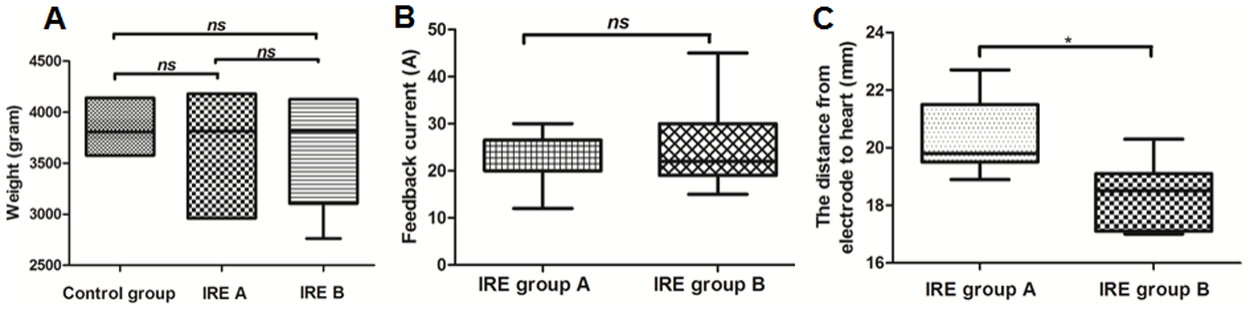
General situation differences between three groups. (A) The differences in body weight between the three groups of rabbits. (B) the difference of feedback current between IRE group A and IRE group B. (C) the difference in distance from the electrodes to the heart between IRE group A and IRE group B.

**Fig 4:**
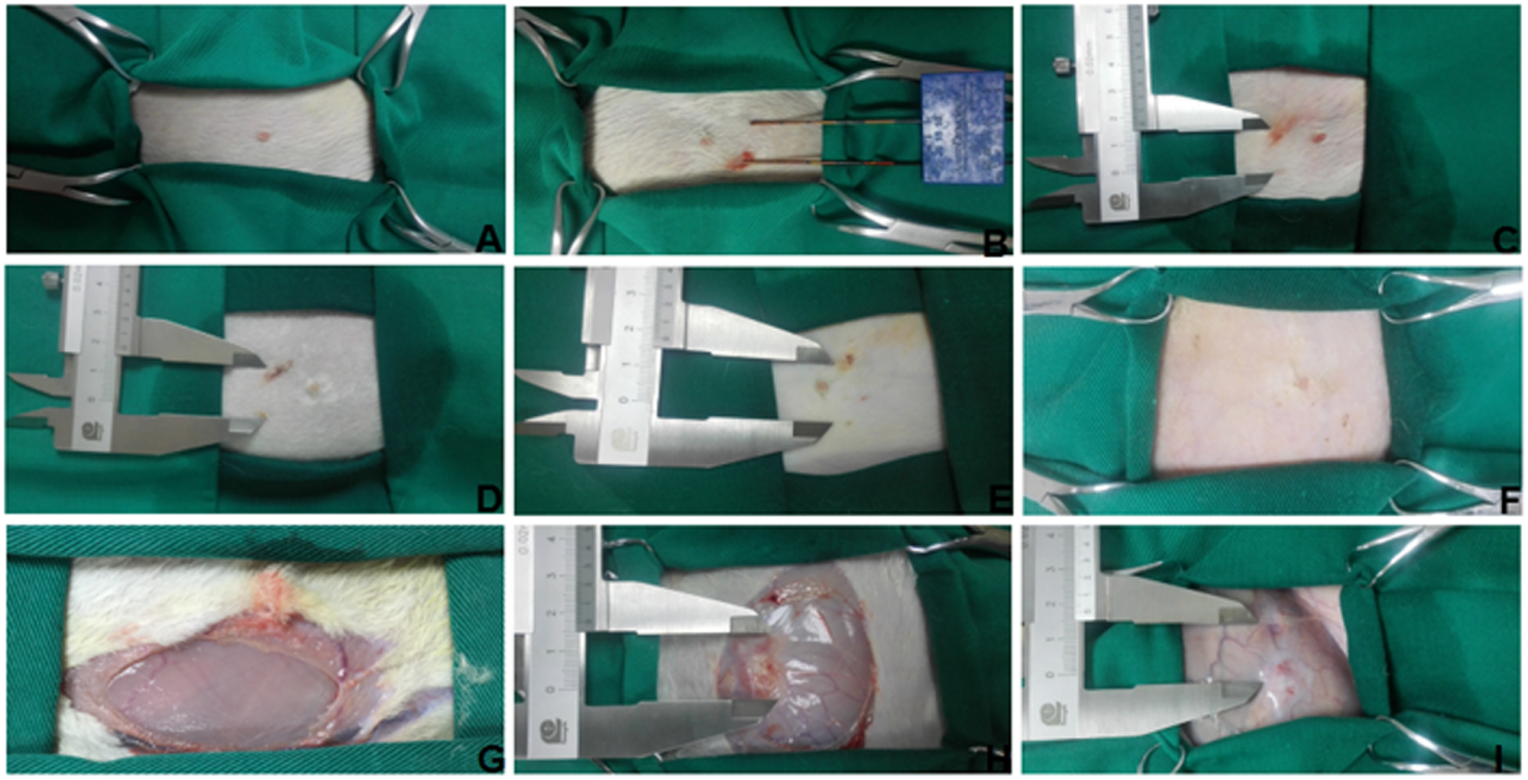
Monitoring breast skin and pectoralis major changes. (A–F) Breast skin. (A) Before IRE; (B) during IRE; (C) Immediately after IRE; (D) 4 days after IRE; E, 7 days after IRE; (F) 14 days after IRE. (G–I) pectoralis major. (G) Before IRE; (H) 4 days after IRE; (I) 14 days after IRE.

The median weight of rabbits in the control group, IRE group A, and IRE group B was 3842 g, 3652 g, and 3644 g, respectively (p > 0.05) (Fig 3A). The median current in IRE group A and B was 22 A and 25 A, respectively (p > 0.05) (Fig 3B). All the rabbits survived to the designated time. Twenty rabbits received electric ablation and were monitored by electrocardiogram (ECG). The median distance from electrode to heart measured by a sterile ultrasonic probe was 21.5 mm in IRE group A and 18.3 mm in IRE group B (p < 0.05) (Fig 3C). No ventricular arrhythmias were detected during the operation in group A. Occasional premature ventricular beats occurred in only 1 rabbit (10%) from group B; fortunately, the rabbit’s postoperative cardiac rhythm was restored to normal. The vital signs of all animals were normal. There was no intraoperative or postoperative sudden death, and we observed no serious bleeding or infection at the percutaneous puncture site. Rabbits from IRE groups A and B underwent the electrical ablation process, while rabbits in the control group experienced the process of anesthesia, puncture, and insertion and removal of the needle without current conveyance (Fig 4A). The breast skin was not burned after ablation; however, subcutaneous hemorrhages were found in 8 rabbits (80%) from group A and 7 rabbits (70%) from group B (p > 0.05) immediately after ablation. These hemorrhages returned to a normal appearance 14 days after operation (Fig 4B–4F). The post-operative autopsy showed that the pectoralis major muscle, located deep below the surface of breast tissue ablation, developed hyperemia at day 1 after ablation, and also developed abnormal gray and white matter at day 14 after ablation (Fig 4G–4I).

### Histological and ultrastructural changes over time

**Fig 5:**
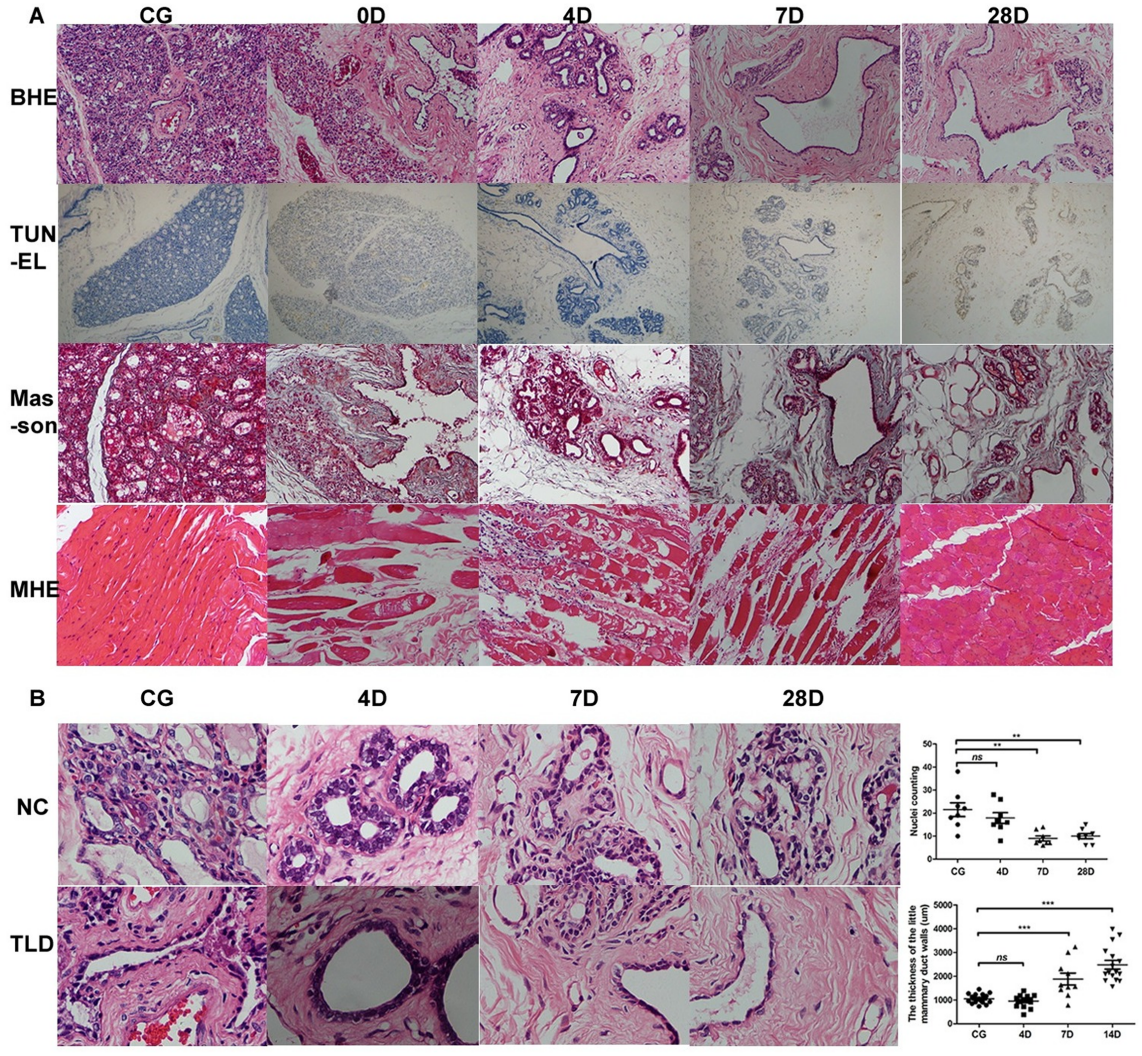
Pathological examinations of control group and IRE treatment groups. (A) HE, TUNEL, and Masson staining in control group and IRE groups, and HE staining of the major thoracic muscle in the control group and IRE group (immediately after ablation and on days 4, 7, and 28 after ablation) (200×). (B) changes in the number of secretory cells in the terminal duct and in the thickness of the little mammary duct walls over time (200×). CG, control group; BHE, HE staining of normal breast tissue; TUNEL, TUNEL staining; Masson, Masson staining; MHE, HE staining of major thoracic muscle; NC, the number of secretory cells; TLD, the thickness of the little mammary duct walls.

Histological changes in the breast after IRE seemed to be relatively mild. Breast ductal epithelial shedding and hemorrhages adjacent to the mammary gland were found; however, there was minimal hemorrhagic necrosis and nuclear condensation immediately after ablation, and mammary gland lobules and terminal ducts were intact. On day 4 after IRE, the integrity of the lobules and the terminal ducts was preserved with some hemorrhaging, a small amount of inflammatory cell infiltration in the interstitial component, and thickened walls of small and medium-sized mammary ducts. Intriguingly, the number of secretory cells was significantly decreased in the terminal duct on day 7 after IRE, coupled with a decrease in hemorrhaging and inflammatory cell infiltration in the interstitial component. Moreover, fibrous tissue hyperplasia and mammary duct thickening was also detected. On day 28 after IRE, there were fewer secretory cells in the terminal duct as well as a notable thickening of mammary ducts and apparent fibrosis in the interstitial structure (Fig 5A). The number of secretory cells in the terminal ducts was counted, and the thickness of the little mammary duct walls was measured at different time points. Compared with the control group, the number of secretory cells decreased significantly on day 7 and day 28 after IRE (21.50 ± 2.98 *vs*. 9.00 ± 1.07, p < 0.01; 21.50 ± 2.98 *vs*. 10.00 ± 1.10, p < 0.01). Similarly, the small mammary duct walls thickened substantially on day 7 and day 28 after IRE (1053.00 ± 48.87 μm *vs*. 1883.00 ± 247.70 μm, p < 0.001; 1053.00 ± 48.87 μm *vs*. 2483.00 ± 186.00 μm, p < 0.001) (Fig 5B). Thoracic major muscle injury was also detected by HE staining. Eosinophilic degeneration of the myocyte was observed under the field of view immediately after ablation; subsequently, striated muscle necrosis, plasma dissolution, muscle fiber fracture, and marked neutrophilic infiltration were observed up to 28 days after IRE. On the last day of observation, we found regeneration of the fibrous tissue between myocytes, residual muscle cells, and scattered muscle tissue around the necrosis region.

Changes in TUNEL staining in the ablation area were relatively mild and durable. TUNEL staining was weakly positive at day 1, and its expression appeared to be stable. At day 28, TUNEL staining was still weakly positive in residual breast tissue (Fig 5A).

**Fig 6:**
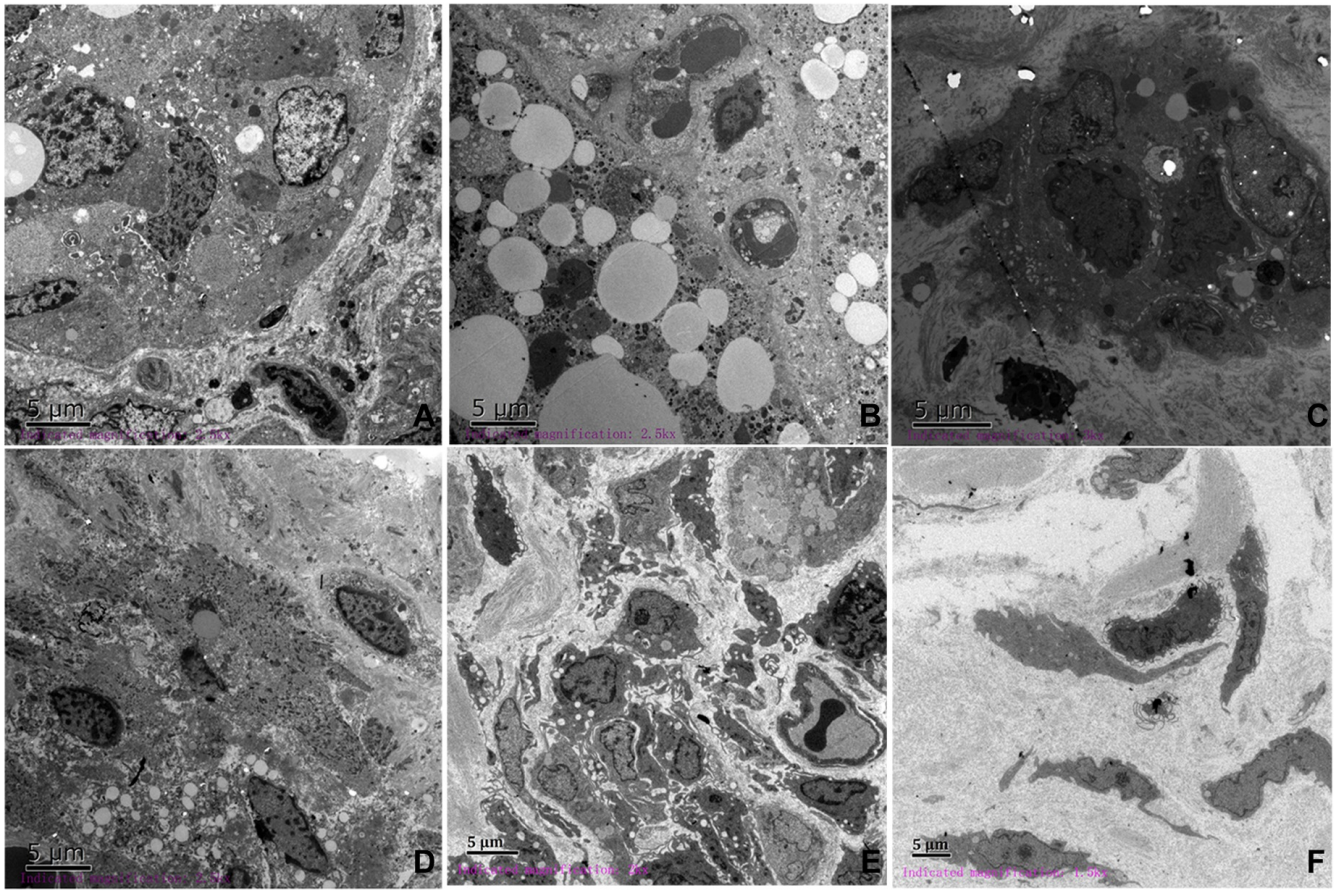
Ultrastructural changes over time (2500×). (A) Normal breast tissue. The nucleus was often filled with euchromatin contained within an intact nuclear membrane. (B) Immediately after IRE, the morphology of the nucleus was slightly irregular, and the chromatin condensed into blocks. (C) 12 hours after IRE, irregular nucleus, invagination of the nuclear membrane, and inflammatory cell infiltration was observed. (D) 24 hours after IRE, chromatic agglutination and nuclear membrane rupture was observed. (E) 4 days after IRE, the formation of autophagosomes in the cytoplasm and a large number of collagen fiber hyperplasia was observed. (F) Normal breast tissue was replaced by collagen fibers.

Observable changes from TEM images were consistent with HE staining in the ablation area. The nucleus filled with euchromatin and enclosed in an intact nuclear membrane was found in normal breast tissues (Fig 6A). Immediately after ablation, the typical changes of apoptosis—chromatin condensation, mitochondria swelling, and endoplasmic reticulum expansion—were detected in mammary gland tissue, and cell membranes were partly disrupted (Fig 6B). From 12 hours after ablation, apoptotic bodies, nuclear dissolution and fragmentation, disintegration of the cytoplasm, and nuclear membrane rupture were observed, and were followed by inflammatory cell infiltration and proliferation of a large number of collagen fibers (Fig 6C–6E). The above changes gradually became more moderate over time and were replaced by the tissue repair process. At day 7, flat and spindle-shaped fibroblasts were scattered in the ablation zone (Fig 6F).

## Discussion

The use of IRE to treat cancers remains rare, including breast cancer. In our study, we employed a rabbit model to systemically study the safety and effects of IRE in breast tissues by observing breast appearance, electrocardiogram, histology, and ultrastructural changes overtime. There was no occurrence of postoperative death, behavioral abnormalities, or serious complications after treatment. These results suggest that IRE may be a promising approach for breast cancer patients who are not suitable for surgical excision.

Concomitant arrhythmias could serve as an imperative index to evaluate the safety of IRE for breast cancer, especially of the left breast adjacent to the heart. Previous studies have shown that two regions form during IRE ablation: a cell death zone close to the applicator surrounded by another zone of tissue with reversible changes in membrane permeability. A myocardium covered by either one of these two zones may lead to a premature action potential and arrhythmia [22]. In our study, only one rabbit in group B had premature ventricular contractions during the ablation process. Consistent with the results of Deodhar et al. [22], a short distance (< 1.7 cm) from the heart and unsynchronized IRE may contribute to cardiovascular events. Therefore, with a lower incidence of fatal events, the implementation of IRE on the right side of the breast seemed to be safer than on the left side. Carrying out synchronized IRE was beyond the scope of this study because of the small size of the rabbits and their relatively fast heart rates. However, another report showed that when the synchronization unit operated incorrectly by delivering pulses in the relative refractory period instead of the absolute refractory period, episodes of ventricular extrasystoles occurred [23]. This indicates that we should remain vigilant when treating a region near the heart. In addition, it is worth noting that most elderly patients have cardiovascular comorbidities and are heavily treated with anthracyclines which were found to cause dose-related toxicity to cardiac cells [24]. Therefore, cardiac function evaluation before IRE ablation, and the application of a synchronization unit and ECG monitor during the operation are strongly recommended.

Breast skin and pectoralis major muscle injuries may be the expansion of the irreversible ablation area to the normal tissue, and thermal energy generated during ablation is non-negligible [25]. In the previous studies, coagulation necrosis [26], skin burns [27–31] as well as pectoralis major injury [31] are common complications detected after IRE simulation. No obvious skin burns were found in group A or group B after the operation in our study. While Noguchi *et al*. summarized long-term outcome and common problem of breast cancer patients treated by radiofrequency ablation and suggested injecting a 5% glucose solution between the skin and tumor, and placing an ice bag over the ablation region [32]. However, if the tumor is too large, necrosis of the internal tumor mass is too advanced, and if there happens to be skin burns after ablation, there may be persistent ulcerated lesions on the breast skin surface, which affects both the esthetic of the breast and the patient's quality of life. Injecting a liquid with low electrical conductivity located between the breast tissue and the major muscle may reduce damage from IRE. This will be addressed in our future studies.

Interestingly, histological examination revealed that the breast tissue underwent only mild injuries, maintaining the integrity of the mammary gland lobules and the end conduits. In contrast, muscle tissue was more severely injured, displaying a wide range of necrosis and apoptosis. This may be partially the result that electrical conductivity of the pectoralis major muscle and fibrous tissue of mammary stroma is higher than normal glandular tissue [33], and different electrical conductivity may trigger different levels of damage in the differentiated tissues. Additionally, given that the greatest effects were observed on the permeability of cell membranes, IRE may preserve the integrity of fibrous tissue and the cytoskeleton without compromising surrounding tissues [34]. This highlights another unique advantage of IRE over other ablation techniques. In our study, the gradually decreased number of secretory cells and thickening of mammary duct lumen could provide further evidence that IRE can induce apoptosis by disrupting cellular homeostasis without destroying the duct and gland structure, making it possible to restore the function and cosmetic appearance of the breast after ablation. Electrostatic shielding may also contribute to protecting breast lobules from electroporation-induced damage. Even in a strong electric field, breast leaflets might be shielded by the highly conductive fibrous tissue that surrounds them. However, during the ablation process, the integrity of cell membranes was destroyed, leading to the redistribution of the electric field and the current loop. Consequently, the current caused only minor damage to the gland and reduced secretory cells in the gland duct. IRE-induced cell death in tissues with different levels of resistance is intriguing and requires further detailed research.

IRE-induced tumor cell death has been confirmed by a number of studies [35, 36], while, though both apoptosis and necrosis have been identified in cells undergoing IRE ablation [37–42], the underlying mechanism remains controversial. Consistent with previous results, both apoptosis and necrosis were observed in our study. Interestingly, the proportion of necrotic cells increased rapidly with an increase in intensity of treatment parameters. In addition, necroptosis, a recently identified form of programmed cell death, presents morphological characteristics similar to necrosis; however, it relies on the activity of receptor-interacting serine/threonine-protein (RIP) kinases rather than caspases. Necroptosis may also be involved in cell death after IRE ablation. Cell death mechanisms are highly complex and will be addressed in our future studies.

Unlike liver tissue, which has strong regenerative capacity after IRE [43], normal breast tissue after IRE was replaced by fibrous tissue without causing any changes in appearance. Fortunately, the integrity of the fat cells around the gland and the vascular structures in the glands were perfectly maintained after IRE. Therefore, the esthetic advantage and the complete ablation rate of IRE supports the use of IRE as an ideal technique for local breast cancer control.

In conclusion, we successfully carried out the safety research of IRE ablation on rabbits and found several advantages of IRE. No obvious complications were observed, and the structure of mammary gland lobules, interstitial components, fat cells, and blood vessels were greatly preserved. IRE ablation is minimally invasive and has better cosmetic outcomes, and is a promising treatment approach for early stage and unresectable breast cancers as opposed to traditional thermal ablation.

Nevertheless, there are limitations to our study. We lacked the ECG data and the ECG synchronization unit during ablation. Although cell death induced by IRE ablation was observed, its specific mechanism was not identified. In future studies, we will evaluate the effects of ECG synchronous units on the physiological activities of the heart during ablation and detect the specific cell death pathway to elucidate the mechanisms of IRE-induced cell death. Moreover, we will observe the effect of ablation on breast tissue under different pre-programmed parameters and the individual predesigned electrode placement modes. Furthermore, we will try to inject a non-toxic liquid with greater resistivity that is easily absorbed by the body into the space between the breast tissue and the pectoralis major muscle to prevent injury. Finally, we will test the efficacy and side effects of ablating larger tumors.

## Supporting information

S1 File ARRIVE guidelines checklist. (PDF)
